# SERS analysis of lubricant additives in the context of technical cleanliness in automotive production

**DOI:** 10.1007/s00216-026-06585-0

**Published:** 2026-06-04

**Authors:** Jannis Gehrlein, Alexander Thomas, Stephanie Kaufmann, Dominik Huber, Natalia P. Ivleva

**Affiliations:** 1 Technical University of Munich (TUM), TUM School of Natural Sciences (NAT, Department Chemistry), Institute of Water Chemistry (IWC), Chair of Analytical Chemistry and Water Chemistry, Lichtenbergstr. 4, 85748 Garching, Germany; 2 Bayerische Motoren Werke Aktiengesellschaft (BMW AG), Petuelring 130, 80788 Munich, Germany

**Keywords:** Surface-enhanced Raman scattering (SERS), Raman spectroscopy, Technical cleanliness, Lubricant analysis, Lubricants, Additives

## Abstract

Filmic contaminations (FCs) from lubricants and other process fluids are an increasingly urgent problem in manufacturing of high-voltage components due to their potential of jeopardizing production steps and, therefore, leading to possible quality issues. In this study, surface-enhanced Raman scattering (SERS) is introduced as a promising candidate for the at-line chemical analysis of FCs with low preparation effort and fast measurements. Simple SERS substrates produced via magnetron sputtering of gold onto aluminum surfaces were tested to successfully analyze model compounds of common lubricant additives 1H-benzotriazole (BTA) and 1,3,4-thiadiazole-2,5-dithiol (DMTD). The determined limit of detection (LOD) for the substrates resulted in a value of 6 × 10^–7^ mol/L for BTA and 9 × 10^–6^ mol/L for DMTD, respectively. Additionally, their shelf-life was determined to assess long-term functionality. It was shown that the substrates could preserve 70% of their functionality after a month of storage under ambient conditions. Afterwards, the method was used for the analysis of three lubricants and it was found that the substrates allowed the identification of BTA additives despite the hydrocarbon matrix, as well as the differentiation of the three lubricants via the used BTA derivative and other additive components. Finally, an extraction method was introduced to transfer an artificial FC from the contaminated surface onto the prepared SERS surfaces and successfully perform chemical analysis of its corrosion-inhibiting additive.

**Graphical abstract:**

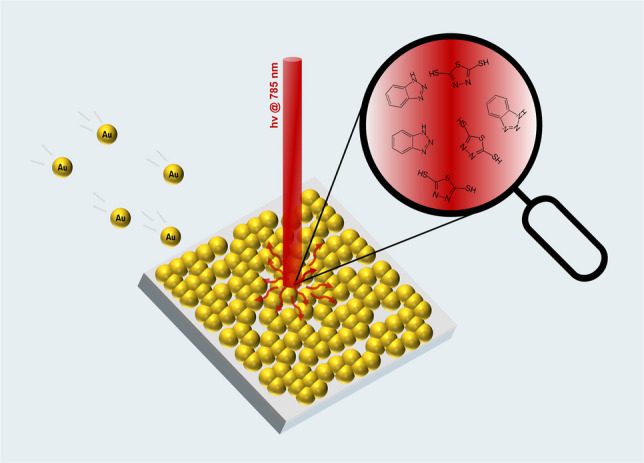

**Supplementary Information:**

The online version contains supplementary material available at 10.1007/s00216-026-06585-0.

## Introduction

In the production of electrified components, e.g., battery or energy module production in the automotive industry, technical cleanliness of the components is of utmost importance. Quality control often focuses on the occurrence of (metallic) particles because of their potential to, among other things, induce short circuits and, therefore, possible thermal runaways [1–4]. However, recent years have increasingly shown the threats of another type of contaminant: filmic contaminations (FCs). They appear as a continuous (non-particulate) film of undesired substance(s) on parts of the surface of the respective workpiece [5]. The broad definition also shows the complexity of the issue and related analysis. While the nature of FCs can vary greatly, they often stem from prior processing steps of the part or from process fluids used in production surroundings. As a result, residues of cooling lubricants, different kinds of oils, fats or waxes, as well as cleaning agents or adhesives can be named [5–8]. Furthermore, noncompliance of cleanliness policies and man-made errors can lead to FCs. Fingerprints or contaminated clothing can be brought up as typical examples for this [5].

All the possible contaminations mentioned share the fact that they can lead to quality issues if not detected. As an example, possible failures of adhesive joints with contaminated surfaces can be named [7, 9, 10]. While the respective error patterns are complex, two main reasons stand out [11, 12]: First, diffusion of the contaminant into the adherend and/or adhesive and, therefore, alteration of the physical properties of selfsame; and second, occurrence of a thin layer of contaminant on the adhesive, physically blocking the substrates from properly connecting. Since adhesive joints can be found increasingly in structure-relevant parts in the automotive industry [13, 14], the absence of FCs is of crucial importance to provide the required quality of the products. Another issue with contaminated components can arise if they are subject to subsequent laser welding, another common process in the automotive industry and other metalworking industries. It is seen as a fact that residues of oils, lubricants, or other organic contaminations can lead to weld cracks and pore formation [6, 15–17]. Naturally, these occurrences impact the integrity of the weld seams and, therefore, threaten the structural stability of the respective components. Additionally, it is reported that organic residue favors the generation of weld sputter during the laser welding process [6, 17]. As mentioned before, the presence of such metallic particles in electrified systems can lead to quality issues of the produced components.

As a result of the high number of possible interferences, robust at-line analysis techniques in industrial surroundings are needed to detect FCs. Furthermore, chemical analysis of the found compounds is of great interest to be able to track contaminations back to their primary source, e.g., process fluids and conservation chemicals. Most industrially used in- or at-line techniques, however, only provide limited chemical information. Among other techniques, fluorescence measurements are the most common. While this method comes with several advantages like the possibility of in-line application, quantitative and space-resolved determination of contaminants, and contact-free measurements [8, 18–20], it does not yield chemical information about the detected compounds. To receive information about the chemical properties of the contaminant, more sophisticated analytical methods are required, which can often only be performed by dedicated laboratories with trained personnel and are connected to high financial expenses. Some examples are thermal desorption-gas chromatography-mass spectrometry (TD-GC-MS), X-ray photoelectron spectroscopy (XPS), or time-of-flight secondary ion mass spectrometry (ToF-SIMS) [5]. As a result of this status quo, a method that can be used at-line or in simple technical cleanliness laboratories on the production site and yields chemical information could help close the existing gap in FC analysis.

A possible solution to this problem is the use of surface-enhanced Raman scattering (SERS). While even today not every detail is understood to the full extent, the general mechanisms of SERS are widely accepted in the scientific community and several book chapters and excellent review articles on the principle of SERS and recent developments and applications are available [21–26]. Because of the depth of this matter, the reader is kindly referred to the mentioned literature for detailed information on the SERS working principles. In this work, SERS substrates were generated by gold magnetron sputtering on easily producible and cheap surfaces consisting of aluminum foil on glass surfaces. Magnetron sputtering in general is a well-known approach for the generation of SERS substrates. Mostly, however, with sophistically crafted base materials [27–32]. This method comes with several advantages for the use of SERS in industrial settings: No chemicals are required for the generation of the substrates, production is simple and fast, and the sputter parameters can be reproduced exactly for each sample. After production, the presented substrates were tested for their SERS properties—including their shelf-life—with common lubricant additive model compounds 1H-benzotriazole (BTA) and 1,3,4-thiadiazole-2,5-dithiol (DMTD).

BTA and its derivatives are broadly used as corrosion inhibitors in metalworking lubricants, especially for copper-containing materials, which is the case for many aluminum alloys [33, 34]. Its frequent use in various (industrial) sectors combined with its low biodegradability can also be deduced from the compounds’ ubiquitous presence in aquatic environments [35, 36]. While DMTD and its derivatives are not as widespread as BTA-related additives, the compounds still play an important role as corrosion inhibitors and metal deactivators for various applications [37, 38]. As a result, analysis of the two model compounds is expected to cover a significant portion of metalworking (cooling) lubricants. Most of the available literature regarding SERS analysis of BTA in real samples focuses on precleaned water (e.g., tap water), with relatively uncomplicated matrices [39, 40]. While there is an excellent paper by Shen et al., proposing an elegant method for the analysis of BTA from transformer oil using SERS, the workflow is more complex and relies on the accessibility of the oil as a bulk fluid, which is not the case for the analysis in technical cleanliness [41]. Most literature on SERS analysis of lubricants is either connected to the field of tribology and, therefore, focuses on the detection of the base components or is used for in situ detection of other long-chain hydrocarbon components [42, 43]. To further advance in this research direction and to show the possibility of SERS measurements of filmic contaminations, the method proposed in this work was also used for the analysis of three industrial lubricants containing BTA or a derivative of its structure.

## Material and methods

### Chemicals

The used solvents for the testing solutions were LC-MS grade LiChrosolv water and acetone in analysis grade (≥ 99.8%), both supplied by Merck KGaA, Germany. Model additives were 1H-benzotriazole (BTA, ≥ 99.0%, Sigma-Aldrich Chemie GmbH, Germany) and 1,3,4-thiadiazole-2,5-dithiol (DMTD, 98.0%, Thermo Fisher Scientific, Belgium). Both analytes were dissolved into solutions of 1 × 10^–4^ mol/L (in water for BTA and a 50:50 mixture of water and acetone for DMTD). The used real sample lubricants were Hycut ET 46 (Oemeta Chemische Werke GmbH, Germany), Hysol SL 45 XBB (Castrol Germany GmbH, Germany), and ECOCOOL AFB 300 (FUCHS LUBRICANTS GERMANY GmbH, Germany). From here on, the lubricants will be referred to as L1, L2, and L3, respectively. Irgamet® 39, an isomeric mixture of BTA derivatives, was provided by BASF SE, Germany. The solvent used for extraction experiments was a 9:1 mixture of cyclohexane (≥ 99.5%, also Sigma-Aldrich) and 2-propanol (≥ 98%, VWR International GmbH, Germany).

### Sample preparation

**Fig. 1:**
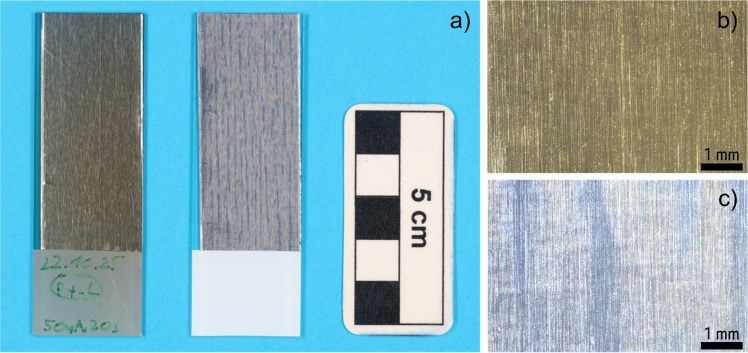
** a** Exemplary images of Al substrates after (left) and before (right) the Au sputter process of 20 s. **b**, **c** Close-up images of the respective surfaces (**b** after and **c** before sputtering)

For the preparation of the SERS measurements, aluminized adhesive tape (Plano GmbH, Germany) was pasted onto conventional microscope slides (VWR) and the aluminum surfaces were cleaned by rinsing with water and acetone. Next, gold was sputtered onto the samples using an EM ACE200 sputter coater (Leica Microsystems GmbH, Germany). A sputter current of 50 mA, an argon working pressure of 4 × 10^–2^ mbar, and a distance between target and substrate of 40 mm were used. Before the sputter process, the chamber was purged with argon and 5 s of pre-sputtering was conducted. From parameter optimization, a sputtering duration of 20 s was found to yield the highest SERS signals and, thus, applied in all experiments. The results for the optimization of this parameter can be found in the next section. Additionally to the optimization of the sputtering parameters, the determination of the LOD for the prepared substrates in combination with the respective analytes was performed. For these experiments, the concentrations of the analyte solutions were lowered stepwise to yield a linear calibration curve. Afterwards, the intensity value of the blank was added to three times the standard deviation and the LOD was obtained after solution of the resulting linear equation [44]. For the shelf-life tests, the samples were then stored in the dark and under ambient conditions (21 °C, 42% relative humidity). Images of exemplary surfaces can be seen in Fig. 1.

For parametrization, LOD- and shelf-life experiments, 10 µL of BTA or DMTD solution with a concentration of 1 × 10^–4^ mol/L was applied on a gold-sputtered surface using a mechanical pipette. After evaporation of the solvent overnight, SERS measurements of the analytes were performed. Experiments with all lubricants, as well as Irgamet® 39, were performed in a similar manner with an analyte volume of 2 µL. For these two sample classes, excess liquid was removed using a TX761MD polyester cleanroom swab (Texwipe, USA). This way, a visually homogeneous, more realistic contamination film was received. Measurements of these analytes were performed directly after preparation of the samples. For the extraction experiments, first, 2 µL of L1 was pipetted onto a blank Al surface. After 5 min of residence time, the liquid was extracted using the previously described extraction solvent. For this, 1 mL of the solvent was used in combination with a polyester swab to extract the cooling lubricant from the surface. The solvent was then evaporated in a beaker until only a highly viscous residue was left. Afterwards, the residue was transferred from the beaker using 50 µL of the extraction solvent. In the last step, the 50 µL was placed on the respective Au surface using a mechanical pipette. This procedure was carried out three times for each sample to receive more reliable results.

### Raman and SERS experiments

SERS measurements of the samples were performed using a LabRAM Aramis Raman spectrometer (HORIBA Europe GmbH, Germany) with a 50× objective (MPlan N, numerical aperture, NA = 0.75, Olympus, Japan) and a 600 g/mm diffraction grating. Measurements were performed in 3 × 3 grids with nine individual acquisitions and a distance of 50 µm between measurement points. They were conducted on the analyte residue as centrally as possible to avoid so-called *coffee ring effects* which might influence the Raman signals due to analyte agglomeration. This was done using the focused beam of an internal light source of the Raman system. In the measurements of the extraction experiments, no defined circle of residue was observable because of the low surface tension of lubricant and solvent. As a result, the measurement grids were placed in random positions of the covered area on the surfaces. Bulk spectra were acquired the same way; however, single spectra were obtained instead of grids. In total, three laser wavelengths were tested for their SERS enhancement capabilities on the gold substrate, 473 nm, 633 nm, and 785 nm. The results can be seen in the Supplementary Information (SI) in Fig. S1. The quality of the received spectra decreased in the following order: 785 nm > 633 nm > 473 nm, which corresponds to the expected photoelectrical properties of gold nanostructures. Final SERS spectra of BTA and DMTD samples were acquired with a 785-nm laser and a power of 10.2 mW (at the sample) with an acquisition time of 5 s. Lubricant and additive samples were measured with the same general Raman parameters with an increased acquisition time of 20 s except for L3 for which 5 s was maintained. The spectra, mean intensities, and standard deviations depicted in this work were calculated from the average spectra of the measurement grids. The exact parameters for the different experiments are shown in the SI in Table S1.

All raw spectra in this work were treated with a baseline correction algorithm using asymmetrically reweighted penalized least squares smoothing (arPLS) to improve spectral quality and simplify insights [45]. Measurements that led to saturation of the detector due to local fluorescence effects were omitted from the results. In total, this affected 2.9% of the 5350 spectra acquired for this work. Normalization was performed by dividing data points by the most intense signal of the spectrum. This signal is mentioned in the descriptions of the respective graphs.

### Surface characterization

Surface characterization was performed by focused ion beam scanning electron microscopy (FIB-SEM) using a Crossbeam 550 with Gemini 2 electron optics (Carl Zeiss AG, Germany). Secondary electrons were detected with an in-lens detector.

## Results and discussion

### Parameter optimization

**Fig. 2:**
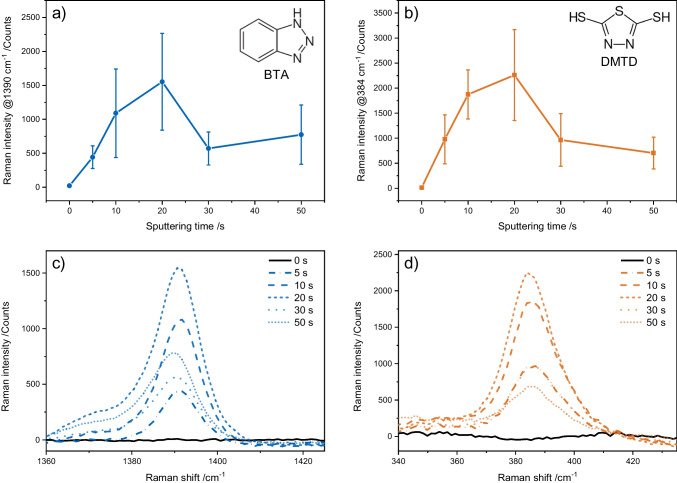
Average Raman signal intensity with varying sputtering times for BTA (**a**, signal at 1390 cm^−1^) and DMTD (**b**, signal at 384 cm^−1^), respectively. Both signals were chosen because of their prominence in the spectra. **c**, **d** Sections of the respective signals of the SERS spectra of **a** and **b**. Mean intensities and standard deviations were calculated from the average spectra of 12 measurement grids obtained from 4 analyte solution droplets. For each average spectrum per grid, 3 × 3 individual spectra were acquired. The used volume and concentration of the solutions before evaporation were 10 µL and 1 × 10^–4^ mol/L

To maximize SERS signals of the tested analytes, it was necessary to perform the sputtering process with varying sputtering durations for the optimal configuration of gold nanostructures on the aluminum surface. The optimization was performed via signal maximization for both BTA and DMTD using the corresponding signals at 1390 cm^−1^ and 384 cm^−1^, respectively. The assignment of these signals to their vibration modes is discussed in the next subchapter. The result of this series of experiments can be seen in Fig. 2.

**Fig. 3:**
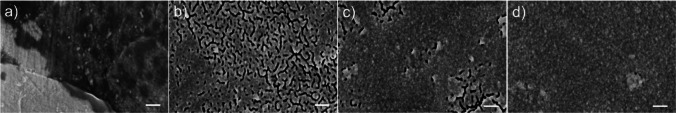
Representative SEM images of prepared surfaces with sputtering times of 0 s (**a**), 10 s (**b**), 20 s (**c**), and 30 s (**d**). A “groove-” or “valley-” like structure of the surface can be seen with semi-long sputtering times in **b** and **c** before a continuous film is formed in **d**. The white bar in the bottom right corner corresponds to a length of 100 nm

Figure 2 shows a clear trend for the influence of sputtering time on both tested analytes. With no gold sputtered onto the surface (0 s), no Raman signals of either analyte can be detected with the used analyte concentration and measurement parameters. Starting with a sputtering time of 5 s, first signals emerge and with longer sputtering durations, an increase in average signal intensity can be seen. The maximum is reached at a sputtering duration of 20 s with an average Raman intensity value of 1553 ± 714 counts for BTA and 2261 ± 907 counts for DMTD. For 30 s, the intensity rapidly decreases to roughly half of the maximum value and does not change significantly for a sputtering duration of 50 s. This trend agrees with literature and can mainly be explained by the different formation of hot spot areas for SERS enhancement depending on different gold film thickness [25, 28, 29]. For better confirmability of this hypothesis, representative SEM images of surfaces sputtered with selected sputtering times are depicted in Fig. 3. Additional images are shown in the SI in Fig. S2.

With very low Au film thickness and only partial coverage of the surface, few agglomerations of gold nanostructures (so-called *nano-islands*) emerge, and some analyte molecules can adsorb onto the noble metal structures. Hence, the SERS effect is introduced and low analyte concentration on the surface can be measured. With increasing Au deposition, the density of these nanostructures on the surface increases, leading to a sort of “groove-” or “valley-” like structure (see Fig. 3b and c). As a result, the chance of analyte molecules in the diameter of the laser beam adsorbing onto the gold structure or occupying a hot spot location, a cavity between two or more parts of the nanostructure, increases. Consequently, the intensity of the signals is drastically enhanced. It is important to understand that a few molecules (out of several hundreds of thousands) in the “perfect” hot spot position can make up a significant percentage of the observed Raman intensity in SERS [21, 46]. Therefore, an increased number of available hot spot sites can lead to the observed results of enhanced signal intensity, even if only a few molecules gain access to these. The drop in signal intensity after sputtering durations of 30 s can be explained by the formation of a more continuous Au film (see Fig. 3d). The structure becomes more plane due to filling of the grooves and, thus, the density of hot spot areas decreases [28, 29]. The SERS effect can still be observed with intensities between 500 and 1000 counts but the reduced roughness of the Au nanostructures suppresses high enhancement to a significant degree.

Observing the datapoints of Fig. 2a and b, it becomes apparent that the variance of the values seems relatively high. At a sputtering duration of 10 s, the relative standard deviation of the intensity is 60% for BTA and 54% at 20 s for DMTD. It is suspected that this is a result of the relatively scarce occurrence of hot spots in the Au nanostructure and, more generally speaking, random placement of areas with higher or lower enhancement capabilities. The standard deviation could most definitely be decreased by working with more sophistically crafted SERS substrates; however, this would withdraw the advantage of the method being simple and inexpensive. Additionally, despite the high error margin, almost all single spectra are of sufficient quality for the identification of the analyte, which is the focus of this work. An overview of the reproducibility of the single spectra of this row of measurements is given in Fig. S3 in the SI of this article.

The determination of the LOD for the obtained set of parameters resulted in a value of 6 × 10^–7^ mol/L for BTA and 9 × 10^–6^ mol/L for DMTD, respectively. The corresponding results can be found in the SI in Fig. S4. These LOD values are far below the expected concentrations of the analytes in real cooling lubricant samples (0.1–3.0 wt% for the ones tested in this work, which corresponds to 8 × 10^–2^ mol/L – 3 × 10^–1^ mol/L for lubricants using BTA) and are comparable to reported sophisticated substrate preparation techniques [40]. It must be mentioned that these values might be decreased further by longer acquisition times during the SERS measurements, which would, however, strongly increase the overall duration of the procedure.

### SERS spectra of BTA and DMTD

**Fig. 4:**
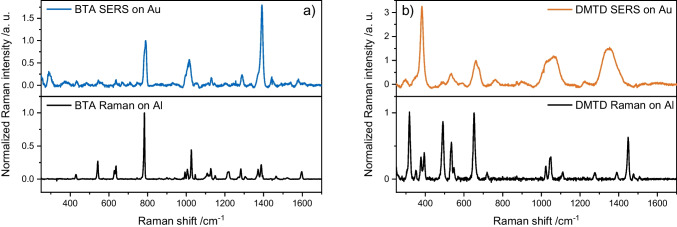
Averaged and normalized exemplary SERS (top) and Raman (bottom) spectra for BTA (**a**) and DMTD (**b**). SERS spectra were calculated from the average spectra of four measurement grids obtained from four analyte solution droplets. For each average spectrum per grid, 3 × 3 individual spectra were acquired. Normalization was conducted by dividing the datapoints by the value of the signal at 790 cm^−1^ for BTA and 660 cm^−1^ for DMTD. Conventional Raman spectra were measured with the same sample procedure as in SERS experiments but with an analyte concentration of 1 × 10^–2^ mol/L for BTA and 2 × 10^–3^ mol/L for DMTD

To further assess the obtained SERS spectra, Fig. 4 displays a comparison of exemplary spectra on a gold-sputtered surface with conventional Raman spectra of the respective compounds at significantly higher concentrations.

It is apparent from Fig. 4 that the effect of the gold substrate on the obtained spectra is significant. For both analytes, shifts of Raman peaks as well as changes in relative intensity of some signals are observable. This, combined with the higher absolute signal intensities of the SERS samples compared to the results of the same analyte concentration on blank aluminum (as shown in Fig. 2), indicates the contribution of both SERS-relevant enhancement factors, *field enhancement* and *chemical enhancement*.

For BTA, three particularly prominent signals in the SERS spectrum in Fig. 4a can be made out at 790 cm^−1^, 1015 cm^−1^, and 1390 cm^−1^. All three signals can also be found with minimal shifts in the conventional Raman spectrum. The assignment to the respective vibration modes results in the signals at 790 cm^−1^ and 1015 cm^−1^ being connected to different benzene ring breathing modes while the signal at 1390 cm^−1^ is attributed to the stretching vibration of the triazole ring [47–49]. The distinct increase in relative SERS intensity of the triazole signal compared to the benzene signals leads to the assumption that the molecule is adsorbed on the gold structures through the lone electron pair of a nitrogen atom of the triazole ring, as has already been suggested by Thomas et al. on a silver surface [48]. Since the field enhancement effect is strongly dependent on the proximity of the analyte molecules, the suggested orientation would allow this functional group to experience even stronger enhancement than the rest of the molecule. Consequently, the relative intensity of the triazole stretching vibration signal is higher in the SERS spectrum compared to the benzene ring signals, different from the conventional Raman spectrum [48].

For DMTD, the exact mechanisms of adsorption on the Au surface are significantly more complex because of the structure of the molecule (thione-thione, thione-thiol, or thiol-thiol tautomer) and various potential adsorption sites (presence of several nitrogen and sulfur heteroatoms). The most prominent signals in the SERS spectrum are located at 384 cm^−1^, 663 cm^−1^, 1062 cm^−1^, and 1353 cm^−1^, where especially the latter two are of particular width. In literature (SERS substrates copper and silver), the signal at 1353 cm^−1^ is attributed to stretching vibration *ν*(C=N) and substituting the respective signal at 1449 cm^−1^ in the conventional Raman spectrum [50, 51]. This suggests the presence of the molecule in the thiol-thiol form. The signals at 663 cm^−1^ (*ν*(C=N)_sym_) and 1062 cm^−1^ (ring stretching mode of the heterocyclic compound) can be seen in both the SERS and conventional Raman spectra [51–53]. The signal at 384 cm^−1^ is attributed to a combination of in-plane deformation vibrations of *δ*(C-S-C) and *δ*(C-S–H) [51]. The prominence of this signal, as well as the presence of the 1353 cm^−1^ signal combined with the peaks of the intact heterocyclic element, leads to the assumption that the molecule is adsorbed on the Au surface in its thiol-thiol form via the nitrogen atoms of the compound.

It must be mentioned that both suggestions for the molecular orientation of the analytes cannot be proven without a sophisticated further analysis, which is not within the scope of this study.

### Shelf-life performance

**Fig. 5:**
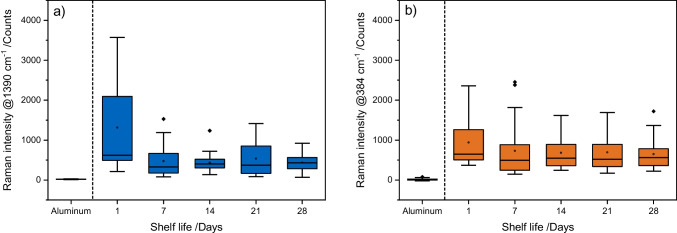
Raman signal intensity with varying shelf-life for BTA (**a**, signal at 1390 cm^−1^) and DMTD (**b**, signal at 384 cm^−1^), respectively. The data points and standard deviations result from the average spectra of 24 measurement grids obtained from eight analyte solution droplets. For each average spectrum per grid, 3 × 3 individual spectra were acquired. The used volume and concentration of the solutions before evaporation were 10 µL and 1 × 10^–4^ mol/L. The datapoints originating in the box plots can be found in Fig. S5 in the SI

To assess the industrial viability of the prepared samples, their shelf-life can be of importance in some scenarios. While the preparation of the surfaces is easy and can be performed with few chemicals and devices, it is still advantage in terms of reproducibility and required preparation time if a high number of surfaces can be produced in one session and then stored under ambient conditions for a longer period of time. This way, a stock of substrates could be prepared for upcoming analysis. To determine the shelf-life of the substrates, five sets of surfaces were prepared with the same parameters at the same time and then tested after increasing storage times. The results of these measurements are depicted in Fig. 5.

Both analytes show similar results in the shelf-life tests. An early measurement after one night of evaporation of the solvent leads to somewhat higher average Raman intensities compared to the following samples. For BTA, the initial median value of the signal at 1390 cm^−1^ is at 617 counts. The experiments after 7 days, 14 days, 21 days, and 28 days show median values of 324 counts, 400 counts, 371 counts, and 429 counts, which correspond to 53%, 65%, 60%, and 70% of the original value, respectively. For DMTD, the average intensities correspond to 76%, 85%, 80%, and 87% of the original intensity value of 645 counts. As a result, it can be said that after a decline within the first week SERS intensity remains stable even after a month of storage under ambient conditions. Also, while an initial drop of 48% in SERS sensitivity seems severe, the remaining potence of the substrate is still strong, especially compared to the blank aluminum surfaces where no analyte signals could be made out.

One possible reason for the decrease in SERS signal intensity with storage time was proposed by Matikainen et al. They reported oxidation and carbon deposition as main factors for substrate degeneration leading to a ~ 70% decrease of SERS signal on Ag films and ~ 90% decrease on Ag nanoparticles, both after 24 h [54]. They argue that while oxidation should not be a problem with Au, carbon deposition might still be of influence. If this is the case in the experiments performed for this work, carbon deposition seems to only have limited impact on the samples, as SERS performance is still available and constant between days 7 and 28. One could then further improve long-term SERS stability by storing the substrates e.g. in a desiccator or in another atmosphere where the surfaces are not subject to moisture, oxygen or hydrocarbon contamination. The fact that the decline in intensity seems to stop before the second measurement also suggests that the surfaces could still undergo some sort of rearrangement within the first hours/days after the sputter process. It has been reported for gold films deposited via electron beam evaporation that initial residual stress of the surface structure decreases by roughly 15% under room temperature storage of 6 days due to surface and grain boundary diffusion effects [55]. This effect is believed to have an influence on the abundance of hot spot areas, in this case on their decline. As a consequence of the two mentioned hypotheses, the results of the first measurements might additionally be improved, if the time between the sputter process and the measurement is reduced further. In theory, both processes can be performed on the same day, if the respective solvent evaporates quickly enough. In this case, however, using water as a solvent hindered quick measurements due to the relatively long evaporation period.

In the BTA samples of 7 days and 14 days, as well as in the DMTD samples of 7 days and 28 days, some outliers higher than the 1.5 × interquartile range between 25 and 75% can be found. Additionally, the range itself (indicated by the whiskers in Fig. 5a and b) shows that the most extreme values within tend to be located on higher intensities than the average and median values. This supports the previously mentioned hypothesis of several hot spot locations in an otherwise homogeneously distributed SERS enhancement.

### Cooling lubricant analysis

**Fig. 6:**
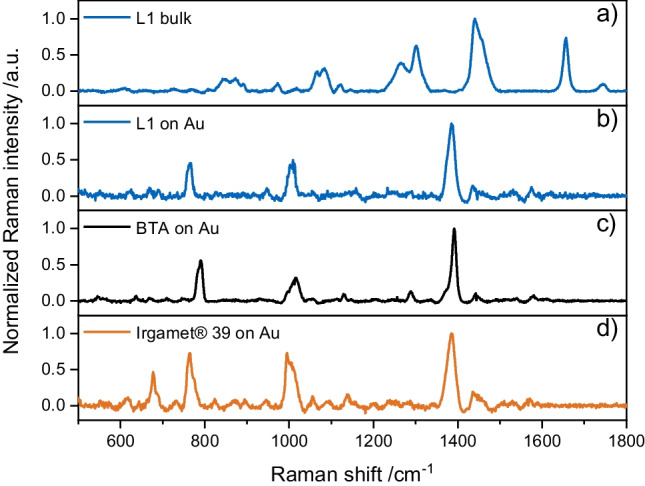
** a** Raman spectrum of cooling lubricant L1 as bulk measurement, **b** average spectrum of the same compound on Au surface, **c** exemplary average BTA spectrum on Au, and **d** average spectrum of Irgamet® 39 on Au. Spectrum **a** was acquired as a single spectrum, while **b** and **d** were calculated from the average spectra of three measurement grids obtained from three (four for **c**) analyte solution droplets. For each average spectrum per grid, 3 × 3 individual spectra were acquired. Normalization was performed by dividing data points by the value of the signal at 1441 cm^−1^ for the bulk and 1385 cm^−1^/1390 cm^−1^ for the SERS spectra

After showing that the produced substrates can enhance Raman signals of relevant cooling lubricant additives in simple matrices and remain usable over a period of at least 30 days, the transfer to real lubricant samples is the next step in the establishment of the method. First, a multifunctional lubricant used for drilling, grinding, thread cutting, and other metal working applications was investigated with the Au-sputtered surfaces. The results are displayed in Fig. 6. For comparison, average SERS spectra of sample additive BTA and real additive Irgamet® 39 are also depicted.

First, it is worth analyzing the Raman signals of the bulk compound itself (Fig. 6a). The most prominent signals can be found at 873 cm^−1^, 973 cm^−1^, 1084 cm^−1^, 1266 cm^−1^, 1301 cm^−1^, 1441 cm^−1^, 1658 cm^−1^, and 1744 cm^−1^. All eight peaks are characteristic of natural oil and fat compounds and can also be found in edible or cooking oils [56–58]. The first two, at 873 cm^−1^ and 973 cm^−1^, can be assigned to skeleton vibrations of saturated fatty acids and their methyl esters [58]. The next five signals stem from various combinations of carbon-carbon or carbon-hydrogen vibrations which are further described in respective literature [56–58]. The signal at 1744 cm^−1^ is assigned to the C=O stretching vibration of the fatty acid methyl ester [57]. This result is not surprising, as nowadays many lubricants are based on natural oils [59].

After application of the lubricant onto the Au-sputtered surface, transformation into a thin film using a polyester swab, and subsequent Raman measurement, a completely different spectrum is obtained (Fig. 6b). Only three signals are now present, at 767 cm^−1^, 1009 cm^−1^, and 1385 cm^−1^, none of which could be found in the bulk spectrum of the lubricant (Fig. 6a). It quickly becomes apparent that this spectrum is similar to pure BTA on Au which, for comparison, is depicted in Fig. 6c. The main difference is a slight shift of the three signals: the 790 cm^−1^ signal in BTA can now be found around 767 cm^−1^ in the lubricant spectrum, the BTA signal at 1015 cm^−1^ is now at 1009 cm^−1^, and the former signal at 1390 cm^−1^ is now present at 1385 cm^−1^, respectively. From the safety data sheet (SDS) of L1, it becomes clear that the used corrosion inhibitor in the product is not BTA itself but a mixture of derivatives of the compound that come with a voluminous hydrophobic extension attached to the triazole ring, most likely for better solubility in the base oil. Additionally, a methyl group is added to the benzene ring, resulting in a tolyl-structure [60]. It must be noted that the provided European Community number of the used additive allows a mixture of structural isomers of this compound, where the methyl group can be found on three of the four benzene ring positions and the hydrophobic N,N-bis(2-ethylhexyl) group can be connected via 1H-methylamine or 2H-methylamine in respect to the triazole ring [61]. One registered trade name of a multi-component mixture consisting of these compounds is Irgamet® 39. The respective molecular structures are shown in comparison to BTA in Fig. S6 in the SI [61, 62].

**Fig. 7:**
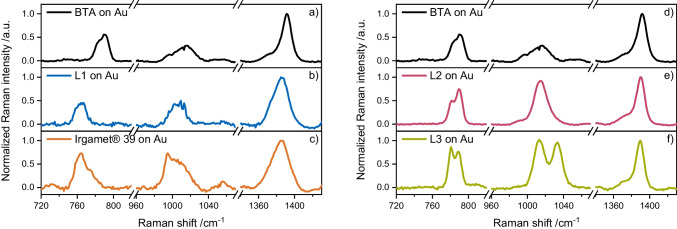
** a**–**c** Sections of the SERS spectra from Fig. 6 in the ranges of the signals at 767 cm^−1^/790 cm^−1^, 1009 cm^−1^/1015 cm^−1^, and 1385 cm^−1^/1390 cm^−1^ for BTA, L1, and Irgamet® 39, respectively. **d**–**f** Corresponding sections for BTA in comparison to lubricants L2 and L3. Mean spectra were calculated from the average spectra of three measurement grids obtained from three (four for **a** and **d**) analyte solution droplets. For each average spectrum per grid, 3 × 3 individual spectra were acquired. Normalization was performed by dividing datapoints by the value of the signal at 1385 cm^−1^/1390 cm^−1^ for all spectra

After repetition of the experiments with Irgamet® 39, the spectrum depicted in Fig. 6d was obtained. It is apparent that the red-shifted signals at 767 cm^−1^, 1009 cm^−1^, and 1385 cm^−1^ are present like in the thin film spectrum of the lubricant (Fig. 6b). Only the Irgamet® 39 signal of the lubricant peak at 1009 cm^−1^ is not found at the exact same wavenumber. This, however, seems to be simply a matter of spectral quality. The respective sections of BTA, lubricant, and additive spectra are depicted in Fig. 7a–c for closer inspection.

The shift of the 767 cm^−1^ and 1009 cm^−1^ signals can most likely be explained by the methyl group attached to the benzene structure. Since these signals are characteristic of the benzene ring breathing mode, a shift in the spectrum is reasonable due to the difference in molecular structure. For the signal at 1385 cm^−1^, the influence of the 1H-methylamine-N,N-bis(2-ethylhexyl) group is most definitely the driving factor towards the observable difference in the Raman shift. The only remarkable difference between the SERS spectra of the lubricant and the additive (Fig. 6b and d) is a signal at 677 cm^−1^ which is not observable in the lubricant. While the origin of this peak is unknown, one possible explanation is that it also stems from the former benzene ring breathing mode with the methyl group on a different position of the benzene ring. As shown in Fig. S6, Irgamet® 39 does not come with a fixed position of this functional group and is, therefore, present as a mixture of several structural isomers. Since this signal cannot be made out in the lubricant spectrum, it is assumed that the relative contributions of individual isomers differ between the two products and the (surface) concentration of the isomer in question might be below the LOD of this system for the lubricant. However, complete resolution of this matter is not possible, since both SDSs do not provide information on the concentration ratios of the additive isomers [60, 62]. Additionally, it must be kept in mind that the method does not allow direct conclusions on the composition of the isomers, as several isomers might lead to similar signals and differences in the adsorption capabilities of the isomers onto the substrate cannot be ruled out. The absence of any other signal in the spectra of lubricant and additive also leads to the hypothesis that (1) the additive is also adsorbed via the triazole ring, as suggested with BTA, and (2) the SERS effect quickly diminishes towards the hydrophobic part of the molecule (most likely due to the increasing distance from the Au surface), as no signs of aliphatic hydrocarbon signals can be found in the observed sections of the spectra, similar to BTA.

To confirm the positive results of the lubricant experiment, measurements were repeated with two additional automotive metalworking lubricants. The results are shown in Fig. 7e and f. It is apparent that characteristic signals of BTA can be found in both spectra. Especially for L2, the BTA signals at 790 cm^−1^, 1015 cm^−1^, and 1390 cm^−1^ are distinctively present. These signals are not deviating from the pure BTA signals; in contrast to the spectrum of L1 in Fig. 7b, even slight shoulders apparent in the 790 cm^−1^ and 1390 cm^−1^ signals of BTA can be made out in L2. The SDS of the product states that BTA is used as an additive in the mixture, which coincides with the obtained SERS spectra [63].

For L3, the obtained spectrum is more complex. While the three discussed signals are present, additional peaks can be observed in close proximity, especially around 790 cm^−1^ and 1015 cm^−1^. First, a new signal at 780 cm^−1^ is now apparent and of similar intensity to the one at 790 cm^−1^. Second, next to the BTA signal at 1015 cm^−1^, a signal at 1034 cm^−1^ of similar intensity can be made out. Again, the European Community number of pure BTA is stated in the SDS of the product as an active ingredient [64], which corresponds to the observed signals. Therefore, the additional signals most likely do not stem from a structural derivate of BTA, different to L1. Since several other additives are present in the product (e.g., not directly specified alkanolamines and isothiazolinone derivates), it is suspected that the signals either stem from these (possibly adsorbed) additional additives or from reaction products of BTA. Additionally, it must be kept in mind that not all components of a product are necessarily stated in the SDS. While this analysis cannot completely resolve the obtained Raman spectrum, it shows another important capability of the method. Not only can BTA and its derivatives be determined in a complex matrix, but different lubricants can be clearly distinguished from each other. Changes in the structure of the triazole additive regarding additional functional groups or changes in the composition of the lubricant lead to shifts of Raman signals—especially of the ones corresponding to the benzene ring—or result in the emerging of additional signals. Both could be resolved with the presented method for all three tested lubricants. This point is crucial for the application of the method onto real samples, as it not only gives a hint towards the possible type of contamination (e.g., cooling lubricant) but also enables the differentiation between process steps, even on a supplier level, assuming the respective lubricants are known.

It must also be noted that the same experimental procedure with all used lubricants, as well as Irgamet® 39, led to no significant Raman signals when performed on the blank aluminum surface, as can be seen in Fig. S7 in the SI. In L3, a minimal signal is found at 790 cm^−1^. However, because of the extremely low intensity and the absence of other characteristic signals, it is not sufficient for chemical identification.

### Analysis of contaminated aluminum sheets

**Fig. 8:**
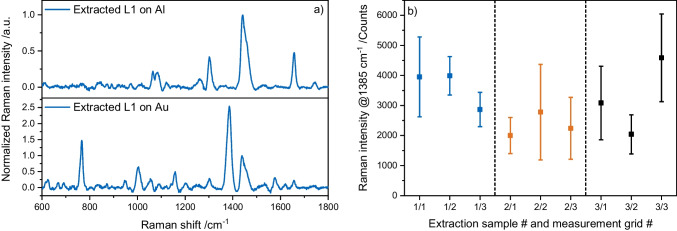
** a** Average Raman spectra of L1 after the extraction procedure onto Al (top) and Au (bottom). Mean spectra were calculated from the average spectra of all nine measurement grids obtained from nine analyte droplets on three gold samples that the extraction process was performed on. For each average spectrum per grid, 3 × 3 individual spectra were used. Normalization was performed by dividing datapoints by the value of the signal at 1441 cm^−1^. **b** Average SERS intensities and standard deviations of the individual extraction samples and 3 × 3 measurement grids on Au surface. Numbers in *x*-axis labels denote sample number before the forward slash and measurement grid number after the forward slash. Blue, orange, and black datapoints show affiliation to the first, second, and third extraction sample, respectively

To further expand the results towards a method which is applicable in an industrial setting, an extraction procedure from surfaces contaminated with L1 was tested. After six extraction experiments, three samples were tested on prepared gold surfaces and three samples on aluminum surfaces, respectively. The resulting average Raman spectra are displayed in Fig. 8a.

The results show that the described extraction process with the chosen hardware and solvent mixture works as intended. The average spectra result in a good signal-to-noise ratio. In both average spectra, most or all of the discussed natural oil signals at 873 cm^−1^, 973 cm^−1^, 1084 cm^−1^, 1266 cm^−1^, 1301 cm^−1^, 1441 cm^−1^, 1658 cm^−1^, and 1744 cm^−1^ can be made out, especially the ones with high prominence in the bulk spectrum (1301 cm^−1^, 1441 cm^−1^, 1658 cm^−1^) in Fig. 6a. The significant difference between the results of the two surfaces is the additional presence of the BTA derivative signals at 767 cm^−1^, 1009 cm^−1^, and 1085 cm^−1^ in the spectra acquired on Au. As a result, the extraction process in combination with the prepared Au surfaces not only results in qualitative transfer of the base contamination but also enables obtaining additional chemical information on the contaminant, in this case the included heterocyclic additive. To assess the reproducibility of the method, results of the single measurement grids of the samples are shown in Fig. 8b.

The standard deviation of the results suggests a relatively high error margin of the method with values up to ± 70% for the second measurement grid on the second extraction sample. However, the averaged received SERS signal at 1385 cm^−1^ always has an intensity > 2000 counts which leads to good interpretability of all individual measurement grids. Thus, the results are accepted as adequate for qualitative determination of the exemplary contamination. Also, it must be differentiated between the uncertainty of the SERS measurement, which is mostly dependent of the surface, homogeneous distribution of the analyte on selfsame—which can be even less controlled as with aqueous solutions due to the low surface tension of the solvent—and the error induced by the manual extraction process. To further investigate this matter, the relative standard deviation of the 1441 cm^−1^ signal on Al of all 9 measurements was determined (= ± 65%) and compared to the same value for the 1385 cm^−1^ signal on Au (= ± 29%). This result suggests that combined uncertainty of the manual extraction and the distribution of the analyte on the surface has a considerably high impact compared to the uncertainty from the SERS measurement. For the extraction process, this could mean that in one of the two steps, the transfer of the contamination from the contaminated surface into the beaker, as well as the transfer of the extract from the beaker onto the testing surface, the compounds were not transferred in a quantitative manner. Additionally, improvements in the deposition of the extracted contamination onto the testing surface must be made to (1) obtain a more homogeneous and defined distribution on the surface, and (2) make sure that the same amount of solvent is transferred in every process without varying evaporation. In conclusion, the proposed extraction method is deemed not ready for quantitative determination of contaminants yet, at least not without further improvement. However, it is applicable for the reliable qualitative analysis of lubricant additives by SERS.

## Conclusion

In this study, simple and reproducible SERS substrates were generated via magnetron sputtering of gold onto aluminum surfaces. Sputtering duration was optimized towards maximum SERS enhancement of analytes BTA and DMTD with an optimum reached after a sputtering time of 20 s. For these parameters, the LOD of the substrates was determined to be 6 × 10^–7^ mol/L for BTA and 9 × 10^–6^ mol/L for DMTD, respectively. Subsequently, the shelf-life of the substrates was tested to assess long-term stability for industrial experimental settings. It was found that the produced surfaces delivered 70% of their original enhancement capabilities for BTA and 87% for DMTD after storage under ambient conditions over a period of 1 month. Afterwards, the developed analysis method was applied to three real cooling lubricant samples. It was shown that common corrosion inhibitor BTA and a derivative could be qualitatively analyzed in all samples due to their affinity to the Au nanostructure despite the complex hydrocarbon matrix. Additionally, it was found that the substrates allowed the differentiation of the three lubricants via the respective BTA derivative and other used additive components, which is a significant step for the applicability of the method in the industrial field. Finally, a simple extraction process was performed with one lubricant to assess the developed method for the analysis of an artificial FC. It was found that qualitative transfer of an exemplary contamination onto the gold substrate was successful and that the surface was able to expand chemical analysis of the contaminant by not only indicating Raman signals of the base oil but also of the included corrosion-inhibiting additive. While there are still ways to further optimize SERS enhancement to enable this method for quantitative measurements of contaminations (substrate generation, extraction process, etc.), the applicability of SERS in the context of FC analysis in technical cleanliness was shown.

## Supplementary Information

Below is the link to the electronic supplementary material.

Supplementary file1 (PDF 7.13 MB)

## Data Availability

Data will be made available on request from the authors.
