# Supplementary material for: SERS analysis of lubricant additives in the context of technical cleanliness in automotive production
Source: Anal Bioanal Chem. 2026 Jun 4;418(16):5303–16. doi: 10.1007/s00216-026-06585-0 (PMC13424648; doi:10.1007/s00216-026-06585-0)
Supplement: Supplementary file 1 — Supplementary file1 (PDF 7.13 MB) [file 216_2026_6585_MOESM1_ESM.pdf]

Supplementary Information (SI)

SERS Analysis of Lubricant Additives in the Context of Technical Cleanliness in Automotive Production

Jannis Gehrlein[a,b]*, Alexander Thomas[a,b], Stephanie Kaufmann[b], Dominik Huber[b], Natalia P. Ivleva[a]*

[a] Technical University of Munich (TUM), TUM School of Natural Sciences (NAT, Department Chemistry), Institute of Water Chemistry (IWC), Chair of Analytical Chemistry and Water Chemistry, Lichtenbergstr. 4, 85748 Garching, Germany

[b] Bayerische Motoren Werke Aktiengesellschaft (BMW AG), Petuelring 130, 80788 Munich, Germany

*Corresponding authors:

Jannis Gehrlein (jannis.gehrlein@bmw.de),

PD Dr. Natalia P. Ivleva (natalia.ivleva@tum.de)

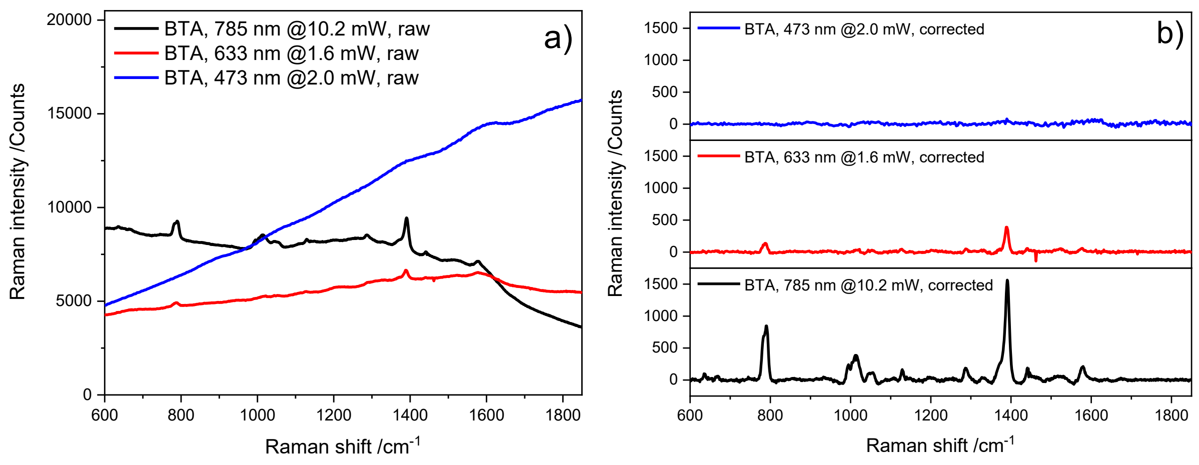

Fig. S1 Average raw (**a**) and corrected (**b**) Raman spectra of BTA on Au with varying laser wavelengths on samples with a sputtering time of 20 s. Mean spectra were calculated from the average spectra of twelve measurement grids obtained from four analyte solution droplets. For each average spectrum per grid, 3×3 individual spectra were acquired. The used volume and concentration of the solutions before evaporation was 10 µL and 1×10^{-4} mol/L. The mentioned absolute values of laser power correspond to 100% of relative possible laser power of the used setup for 633 nm and 785 nm and 50% for 473 nm, respectively

Experiment	Measurements per grid	Number of analyte droplets	Number of measured grids per droplet	Resulting average spectra per parameter
Parametrization	9	4	3	12 (108)
LOD	9	3	3	9 (81)
Shelf-life tests	9	8	3	24 (216)
Lubricants	9	3	1	3 (27)
Extraction	9	9	1	9 (81)

Table S1. Measurement parameters used for calculation of depicted Raman spectra, mean intensities and standard deviations in this work. Each depicted number represents one data point of the respective measurement, i.e. one concentration in the parametrization experiments

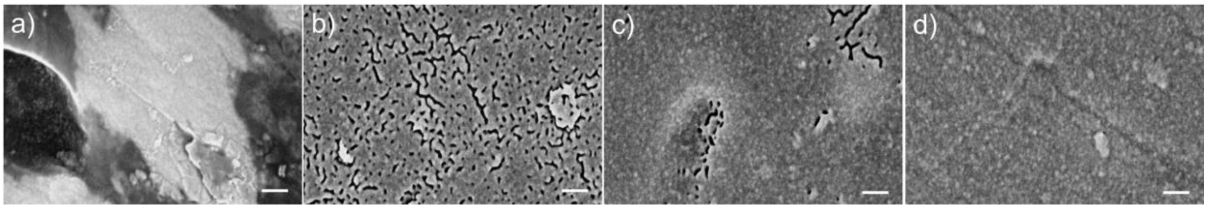

Fig. S2 Additional SEM images of prepared surfaces with sputtering times of 0 s (**a**), 10 s (**b**), 20 s (**c**) and 30 s (**d**). A "groove-" or "valley-" like structure of the surface can be seen with semi-long sputtering times in **b** and **c** before a continuous film is formed in **d**. The white bar in the bottom right corner corresponds to a length of 100 nm

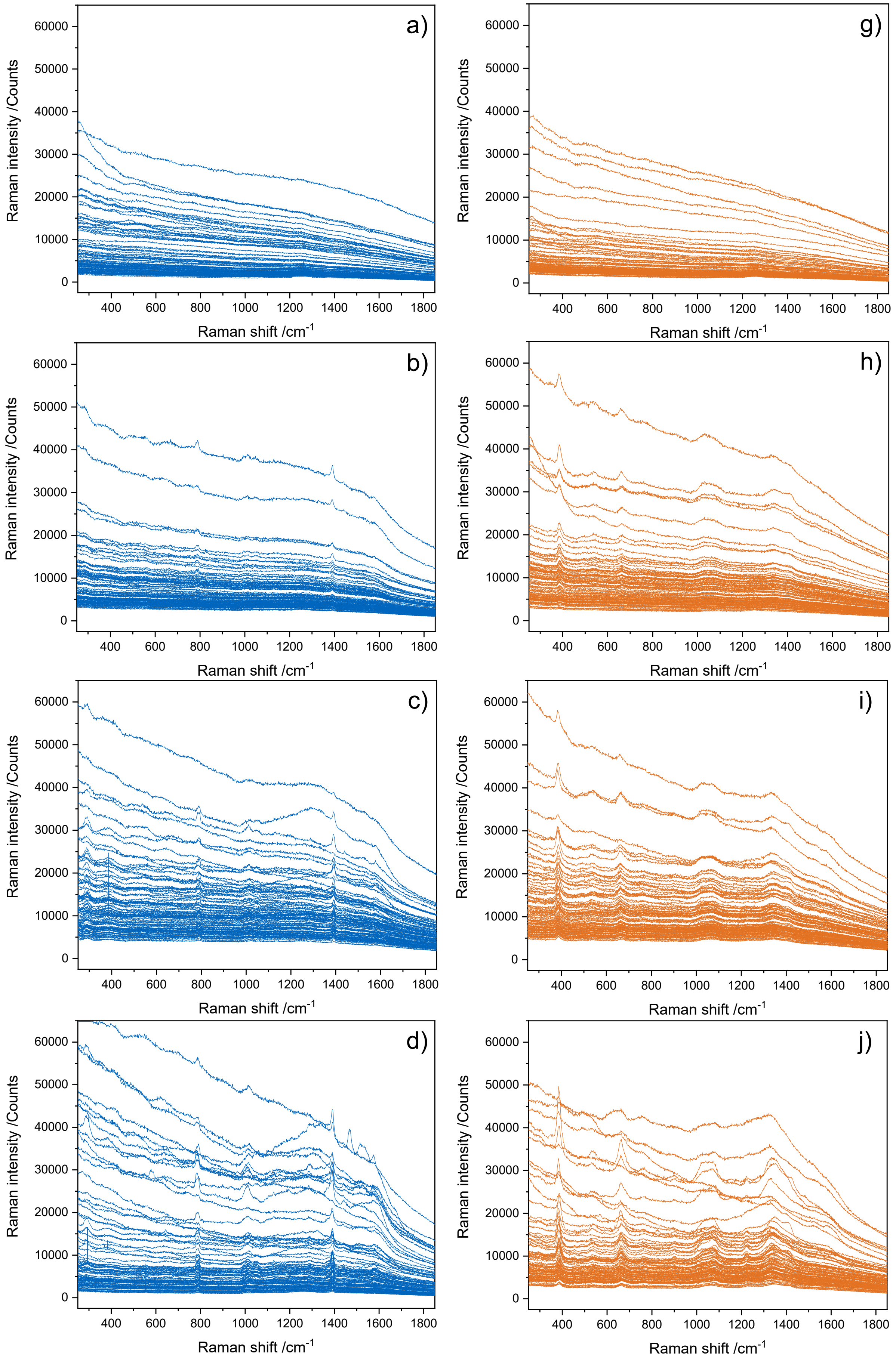
a)
b)
c)
d)
g)
h)
i)
j)
Raman intensity /Counts
Raman shift /cm-1
0
10000
20000
30000
40000
50000
60000
400
600
800
1000
1200
1400
1600
1800

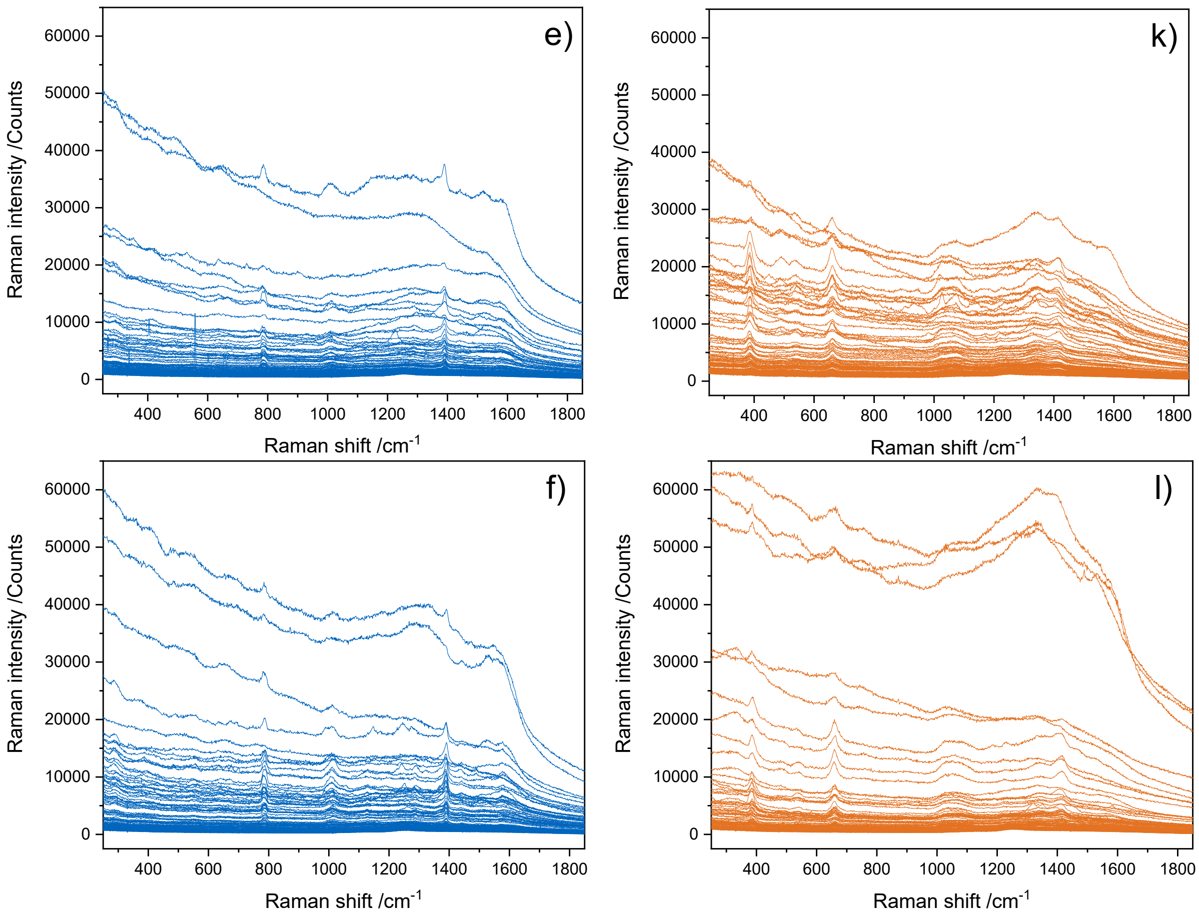

Fig. S3 Single raw Raman spectra of BTA (**a-f**) and DMTD (**g-l**) on Au with varying sputter time of 0 s, 5 s, 10 s, 20 s, 30 s and 50 s (from top to bottom). Spectra that led to saturation of the detector (≥65000 counts) due to fluorescence effects were omitted from the raw data

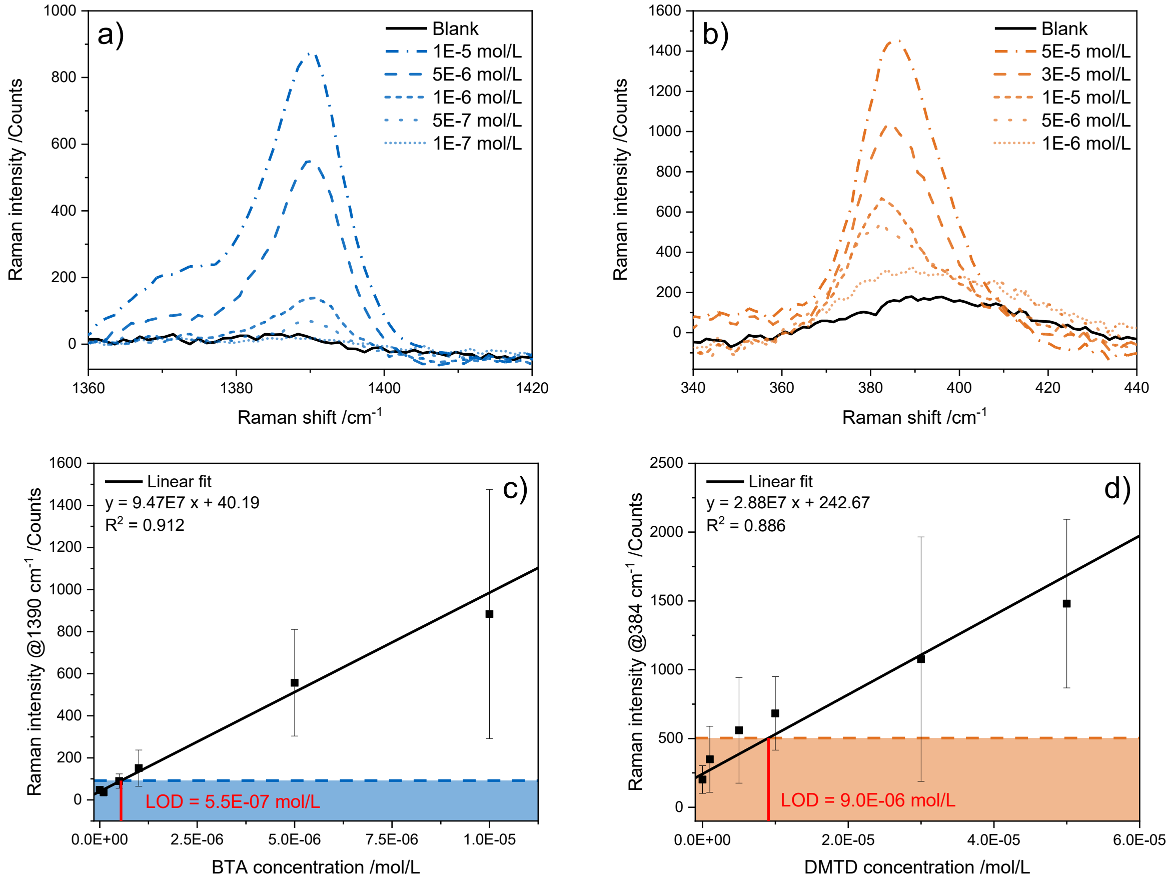

Fig. S4 a and **b**: Sections of average Raman spectra of varying concentrations (n = 6) for BTA (1390 cm^{-1}) and DMTD (384 cm^{-1}), respectively. **c** and **d**: Corresponding linear regressions with equations and LOD values. Mean intensities and standard deviations were calculated from the average spectra of nine measurement grids obtained from three analyte solution droplets (m = 9). For each average spectrum per grid, 3×3 individual spectra were acquired. The used volume of the solutions before evaporation was 10 µL

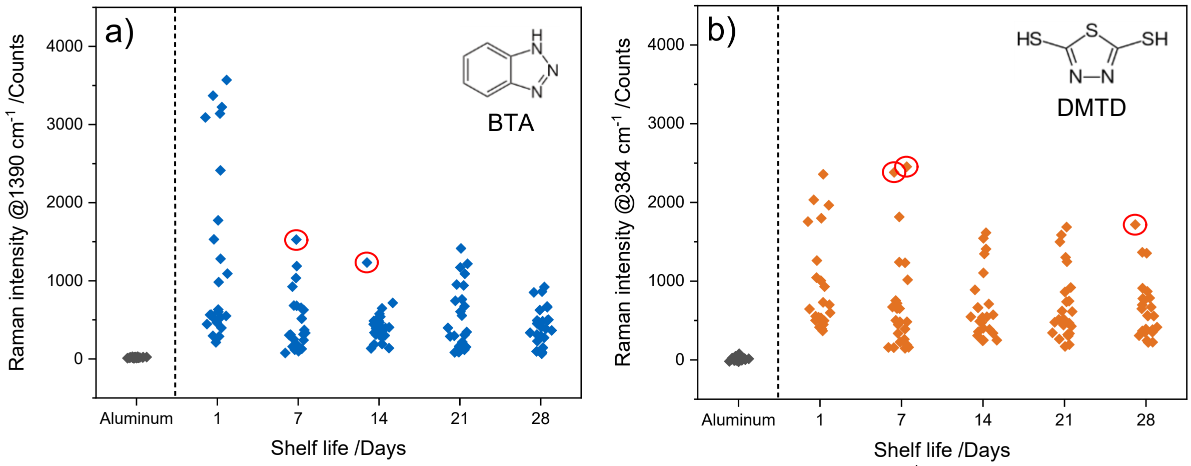

Fig. S5 Raman signal intensity with varying shelf life for BTA (**a**, signal at 1390 cm^{-1}) and DMTD (**b**, signal at 384 cm^{-1}), respectively. Each data point represents an average spectrum of the 24 measurement grids obtained from eight analyte solution droplets. For each average spectrum per grid, 3×3 individual spectra were acquired. Data points marked with a red circle indicate outliers from the 1.5× interquartile range between the 25% and 75% percentile

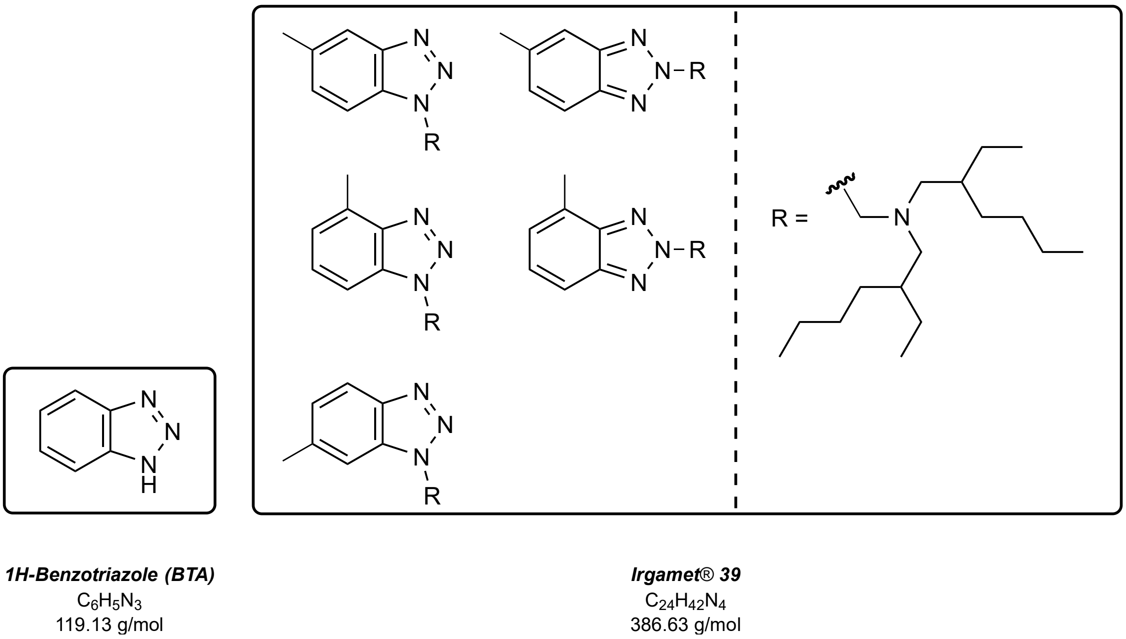

Fig. S6 Molecular structures of BTA and *Irgamet® 39* obtained from the respective SDS [62]

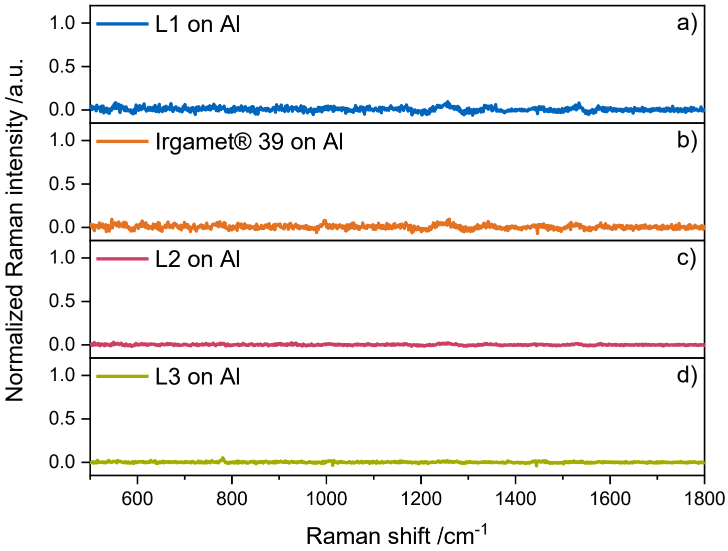

Fig. S7 Raman spectra of cooling lubricants L1 (**a**), L2 (**c**), L3 (**d**) and additive *Irgamet® 39* (**b**) on Al. Mean spectra were calculated from the average spectra of three measurement grids obtained from three analyte solution droplets. For each average spectrum per grid, 3×3 individual spectra were acquired. Normalization was performed by dividing datapoints by the value of the signal at 1390 cm^{-1} / 1385 cm^{-1} of the respective SERS spectrum
